# Health Personality, Resilience, and Activation

**DOI:** 10.1093/geroni/igaa057.2165

**Published:** 2020-12-16

**Authors:** Joseph Kim

**Affiliations:** Iowa State University, Ames, Iowa, United States

## Abstract

The purpose of this study was to identify associations among health personality, resilience, and health activation. Participants included 3907 older adults, 65 and older. Latent variable structural equation modeling with bootstrap sampling estimation was conducted. Significant direct paths were observed between health personality factors and resilience, and in turn, resilience to health activation. The results indicate that older adults who were more health conscientious, more extraverted, and less neurotic about their health were also more resilient. Also, older adults who were more resilient were more health activated. Lastly, older adults who were more health conscientious and more agreeable about their health were more health activated. Resilience had a negative indirect effect on health activation through health neuroticism and health extraversion, and a positive indirect effect through health conscientiousness. Healthcare practitioners should target older adults based on health personality and resilience levels and develop interventions to increase health activation.

